# *Patient readiness* for shared decision making about treatment: Conceptualisation and development of the Ready^SDM^

**DOI:** 10.1111/hex.13995

**Published:** 2024-02-23

**Authors:** Sascha M. Keij, Anne M. Stiggelbout, Arwen H. Pieterse

**Affiliations:** ^1^ Department of Biomedical Data Sciences, Medical Decision Making Leiden University Medical Center Leiden The Netherlands; ^2^ Erasmus School of Health Policy and Management Erasmus University Rotterdam Rotterdam The Netherlands

**Keywords:** patient readiness, questionnaire, shared decision making, treatment decision making

## Abstract

**Introduction:**

Shared decision making (SDM) requires an active role of both clinicians and patients. We aimed to conceptualise patient readiness for SDM about treatment, and to develop a patient questionnaire to assess readiness.

**Methods:**

We used the results of a scoping review and a qualitative study to inform the patient readiness construct. We conducted five additional rounds of data collection to finalise the construct definition and develop the Patient Readiness for SDM Questionnaire (Ready^SDM^) in an oncological setting: (1) longitudinal interviews with patients with cancer during and after a treatment decision‐making process; (2) a pilot study among experts, clinicians, and patients for feedback on the concept and items; (3) a field test among (former) patients with cancer to test item format and content validity, and to reduce the number of items; (4) cognitive interviews with people with low literacy to test the comprehensibility of the questionnaire; and (5) a field test among (former) patients who faced a cancer treatment decision in the last year, to test the content validity of the final version of the questionnaire.

**Results:**

A total of 251 people participated in the various rounds of data collection. We identified eight elements of patient readiness for SDM about treatment: (1) understanding of and attitude towards SDM; (2) information skills; (3) skills in communicating and claiming space; (4) self‐awareness; (5) consideration skills; (6) self‐efficacy; (7) emotional distress; and (8) experienced time. We developed the 20‐item Ready^SDM^ to retrospectively measure these elements in an oncological setting.

**Conclusion:**

We conducted a thorough procedure to conceptualise patient readiness and to develop the Ready^SDM^. The questionnaire aims to provide novel insights into ways to enhance SDM in daily practice.

**Patient or Public Contribution:**

Multiple people with lived experience were involved in various phases of the study. They were asked for input on the study design, the conceptualisation of readiness, and the development of the questionnaire.

## 1 INTRODUCTION

Shared decision making (SDM) is highly recommended for many decisions in clinical practice, and especially those that are preference‐sensitive, that is, decisions where multiple medically reasonable options exist and the patient's values and preferences are important in selecting the best option.^1^ A majority of patients prefer to be involved in decision making.^2^, ^3^, ^4^ However, SDM does not yet frequently occur in daily practice.^2^, ^5^, ^6^, ^7^, ^8^ In the SDM process both the clinician and the patient need to take an active role.^9^, ^10^ For instance, clinicians need to determine the available treatment options, explain that a decision has to be made for which the patient's opinion matters, provide information, and get to know their patients and their preferences.^9^ The active role of patients also consists of various behaviours. During consultations, patients may need to ask questions, express their thoughts and feelings, consider the options, and express preferences. Outside of consultations, patients may need to access information, consider the options, and discuss the decision with significant others.^9^ Patients may struggle with some or all of these SDM tasks, and patients may not always feel ready (i.e., well equipped and enabled) to participate in SDM,^11^ at any one time during the decision‐making process.

In a previous qualitative study, we identified five elements that may constitute *patient readiness for SDM about treatment*.^11^ According to this study, patients may need to (1) understand their role in SDM and have a positive attitude towards involvement; (2) be able to absorb and understand the relevant information; (3) be able to communicate with their clinician and claim space to express themselves; (4) be aware of their goals and values; and (5) be able to consider the different options and have insight into the possible consequences for their personal lives. We were aware that the elements we had identified may not be exhaustive, and that other elements may also play a role.^11^, ^12^, ^13^, ^14^ The extent to which patients feel ready likely fluctuates throughout the decision‐making process. Fluctuations can be caused by various factors, such as the complexity of information,^14^ the communication with the clinician, and the patient's physical and emotional state.^14^, ^15^ Patients may thus feel more or less *ready* to be involved in SDM at different time points during the SDM process (i.e., before, during, in‐between, or after consultations, until a final decision is made). To become more ready for SDM, and therefore enhance SDM in clinical practice, patients may need support during this process. Patient decision aids have been developed to support patients in SDM, and have been demonstrated to help patients by increasing knowledge and clarifying values.^16^ However, these aids do not address other aspects that may be needed for patient involvement in SDM,^12^, ^17^, ^18^ such as a good patient‐clinician relationship and emotional support.^11^, ^17^ Patients may also need to perceive that they have the opportunity and personal ability to be involved, and to consider what is most important to them given the decision they face.^12^ To understand what support patients may need to become more ready for SDM, we first need to fully understand what constitutes readiness. Therefore, our first aim was to conceptualise patient readiness for SDM about treatment.

We also need insights into what elements of readiness patients generally struggle most with. A patient self‐report questionnaire for use in research would allow the identification of the most common challenges patients face in being actively involved in SDM, and of factors that may hinder readiness. Such insights would allow for the identification of general support needs among (subgroups of) patients. To measure readiness, several aspects need to be considered. First, fluctuations in readiness throughout the SDM process make it challenging to meaningfully measure readiness at the start or in the middle of this process, as this could lead to an unreliable indication of a patient's *overall* level of readiness. Therefore, it is most relevant to measure patients' self‐reported level of readiness throughout a decision‐making process shortly after the decision has been made. This way, patients can reflect on how they felt overall throughout the process. Second, the specific aspects of readiness that are most relevant to measure may differ between settings. Therefore, it may be important to focus on one specific setting. In oncology, preference‐sensitive decisions are common,^19^ making SDM highly relevant.^1^ Despite the general preference for the involvement of patients with cancer,^2^, ^20^, ^21^, ^22^, ^23^ the occurrence of SDM is low.^2^, ^5^, ^6^ Involvement may be particularly difficult due to the emotional impact of the diagnosis,^14^ real or perceived time constraints,^14^ and decision complexity.^17^, ^19^ Thus, our second aim was to develop a questionnaire to retrospectively measure patient readiness for SDM about cancer treatment shortly after the decision was made. It is our ambition to raise awareness among clinicians that patients wish to participate in SDM but may not always be ready to be involved. A questionnaire measuring readiness has the potential to provide the necessary evidence on what areas of readiness patient struggle most with, and to detect potential associations with patient‐ and context‐related characteristics, and may thus inform the design of effective support.

## 2 METHODS

### 2.1 Design

We aimed to conceptualise patient readiness for SDM about treatment, and to develop a questionnaire to measure the extent to which patients with cancer felt ready for SDM. We used the COnsensus‐based Standards for the selection of health Measurement Instruments (COSMIN) checklist as a guideline.^24^ Sample sizes were determined based on the recommendations of the COSMIN checklist. To measure patient readiness, we assumed a formative measurement model, that is, the underlying elements define the construct.^25^ The individual items contribute a part of the construct of readiness, and together the items form, rather than reflect, the construct. The elements may, but do not necessarily need to, correlate with each other. For example, patients may have a positive attitude towards involvement, but have difficulties with understanding information about treatment options.

We conducted semi‐structured longitudinal interviews with patients with cancer. We integrated the findings with those of previous studies ^11^, ^14^ and input from the advisory committee. We developed provisional items to measure readiness and conducted a pilot test, a field test, a comprehensibility test, and a second field test to develop the final questionnaire. The Medical Ethical Committee of the Leiden University Medical Center (LUMC) provided exemption for ethical approval of the study protocol (P17.121), according to Dutch Law.

### 2.2 Conceptualisation and item pool creation

In addition to previous research of our group,^11^, ^14^ we wished to further clarify what patient readiness for SDM about treatment entails. A limitation of the previous interview study was the inclusion of patients who faced a decision in the past 6 months, meaning that patients may not have fully remembered how they experienced this process. Further, by interviewing patients at one time point only, we were limited in our understanding of how readiness may potentially change over time. Therefore, we interviewed patients with cancer throughout the decision‐making process. We included patients who were currently facing a treatment decision (i.e., a decision to start, forego, change, or stop cancer treatment). Seven clinicians at LUMC agreed to recruit patients. Patients who were interested, received study information and could contact the researchers or indicate that the researchers could contact them. Patients who agreed to participate and provided informed consent were interviewed at three time points: (1) when they knew a decision had to be made; (2) 1–2 weeks after the decision had been made; and (3) approximately 2 months after the decision had been made. Interviews took place at LUMC or by phone. The semi‐structured interviews were conducted by a female researcher (S. K.) or research assistant (N. v. D.), both trained and experienced in conducting interviews.

Before the first two interviews participants received a short questionnaire about sociodemographic characteristics and how they felt about the decision. At the start of the interview, we described what SDM entails. This description was informed by two models.^9^, ^26^ A summary was placed in front of the participant during the interview (Appendix SA). Patients were asked about what they believe patients need to be able to do to participate in SDM. The interview guides can be found in Appendix SB. Participants received a small present after each interview to thank them for participating. Interviews were audio‐recorded and transcribed verbatim. For the analyses we built upon the coding list and categorisation as developed in our previous qualitative study on patient readiness.^11^ We used Atlas.ti for the analyses. Transcripts were coded by N. v. D. and checked by S. K. Disagreements were resolved in consensus. Codes were categorised thematically by S. K. and N. v. D. Codes and categories were added or renamed when considered necessary, leading to a list of elements of readiness. We sent a document containing a provisional list of (sub‐)elements to the advisory committee members (consisting of clinicians who work in oncology, healthcare communication experts, and oncology patient representatives) and asked them to provide written feedback. We discussed and considered the results of longitudinal interviews, the results of the scoping review^11^ and the feedback provided by the advisory committee. We then defined the provisional list of elements and subelements of readiness. Next, we developed a minimum of four potential items for each subelement. We aimed for items to be short and comprehensive.

### 2.3 Pilot test

We conducted a pilot test among healthcare communication experts, clinicians, and (former) patients. Experts and clinicians were recruited through our network. The (former) patients were members of cancer patient organisations who were involved in our advisory committee. We sent participants a document containing the provisional list of (sub‐)elements, and asked for written feedback on the relevance and completeness of the list. We also asked the experts for feedback on the relevance and completeness of the item pool. Participants could provide their feedback in the provided document. We discussed and considered their feedback and adapted the list of (sub‐)elements and items.

### 2.4 First field test

We included people with a (former) diagnosis of cancer, aged ≥18 years, and able to understand and write Dutch. We recruited participants through a Dutch online panel (kanker.nl). People with a (former) diagnosis of cancer and an interest in participating in research focused on living with cancer can register themselves for this panel. Panel members who met the inclusion criteria were approached once by email. The email was sent by the panel manager and contained a link to the survey. Information about the study was provided on the first page, after which participants provided informed consent by checking a box. Participants were asked about their age, gender, educational background, and diagnosis.

We asked participants for their opinion on the format of the items. For three example items we asked if they preferred items to be formulated as statements (e.g., ‘I understood that it was important to voice my opinion’) or questions (e.g., ‘Did you understand that it was important to voice your opinion?’).

For the remainder of the survey, participants were randomly assigned to evaluate two or three elements, to limit burden. For each element, we showed the subelements we identified so far, and asked participants' opinions on: (1) which subelements were most important for a specific element; (2) if any subelements were not relevant; and (3) if any subelements were missing. We then showed each subelement with the suggested items, formulated as questions, and asked participants' opinions on which items they considered most suitable.

The survey was anonymous. Consequently, we could not identify participants. The survey did further not allow participants to save their answers; participants had to start over if they closed the survey before completion. It is likely that some initiated the survey multiple times. We prioritised including unique responses over having more data and thus retained only fully completed questionnaires, assuming these were data from different participants.

We tested participant preference for response option format using one‐sample *χ* ^2^ tests. The results informed our final choice. The questionnaire was further adapted based on predefined decision rules (Appendix SC) and discussion (S. K. and A. P.).

### 2.5 Comprehensibility test

Two female advisors from Pharos (Dutch Centre of Expertise on Health Disparities) conducted cognitive interviews with adults with low literacy levels. Interviews took place in person or through video calling. The interviewers took notes during the interviews. Participants were shown a draft questionnaire. Participants were asked to read the introduction text and the items aloud and reflect on what they believed it meant. They were asked if the introduction text and items were clear or should be rephrased, and if so, they were asked for suggestions for improvement. For certain items, participants were shown multiple formulations and asked to select the clearest one. Participants were also asked about the clarity of the response scale and how they would experience filling in the questionnaire. Based on the results of the interviews, the advisors from Pharos provided suggestions to improve the comprehensibility of the questionnaire. We adapted the questionnaire based on these suggestions.

### 2.6 Second field test

We conducted a second field test to assess the comprehensiveness and relevance of the 20 provisional items of the questionnaire and to test the response scales. Participants were recruited through the same online panel as in Field test 1. Only people who had been diagnosed with cancer between 2021 and 2023 were approached. Recruitment and informed consent were conducted as in Field test 1. Additional recruitment was conducted at the LUMC. Ten medical specialists and three nurses agreed to recruit patients. Patients who were interested received study information, a consent form, and a questionnaire on paper. The informed consent form and questionnaire could then be handed in at reception or sent back to the researchers.

We included people who had faced a cancer treatment decision in the past year, and were aged ≥18 years and able to understand and write Dutch. Participants were asked about their age, gender, educational background, and diagnosis. Then, participants were asked to complete the provisional Patient Readiness for SDM Questionnaire (Ready^SDM^). Participants were randomly assigned to fill out the questionnaire with either the provisional four‐point response scale or a five‐point response scale included for comparison. We compared the results of the four‐point against the five‐point scale, to ensure the provisional four‐point scale did not show lower variability or higher ceiling effects.

Participants recruited through the online panel were further asked whether any items were missing or redundant, and how they experienced filling in the questionnaire. Results were analysed based on predefined decision rules (Appendix SC). Decisions were made in consensus (S. K., A. P., A. S.) and the final version of the questionnaire was translated to English by a translation agency through forward‐backward translation.

## 3 RESULTS

**Figure 1:**
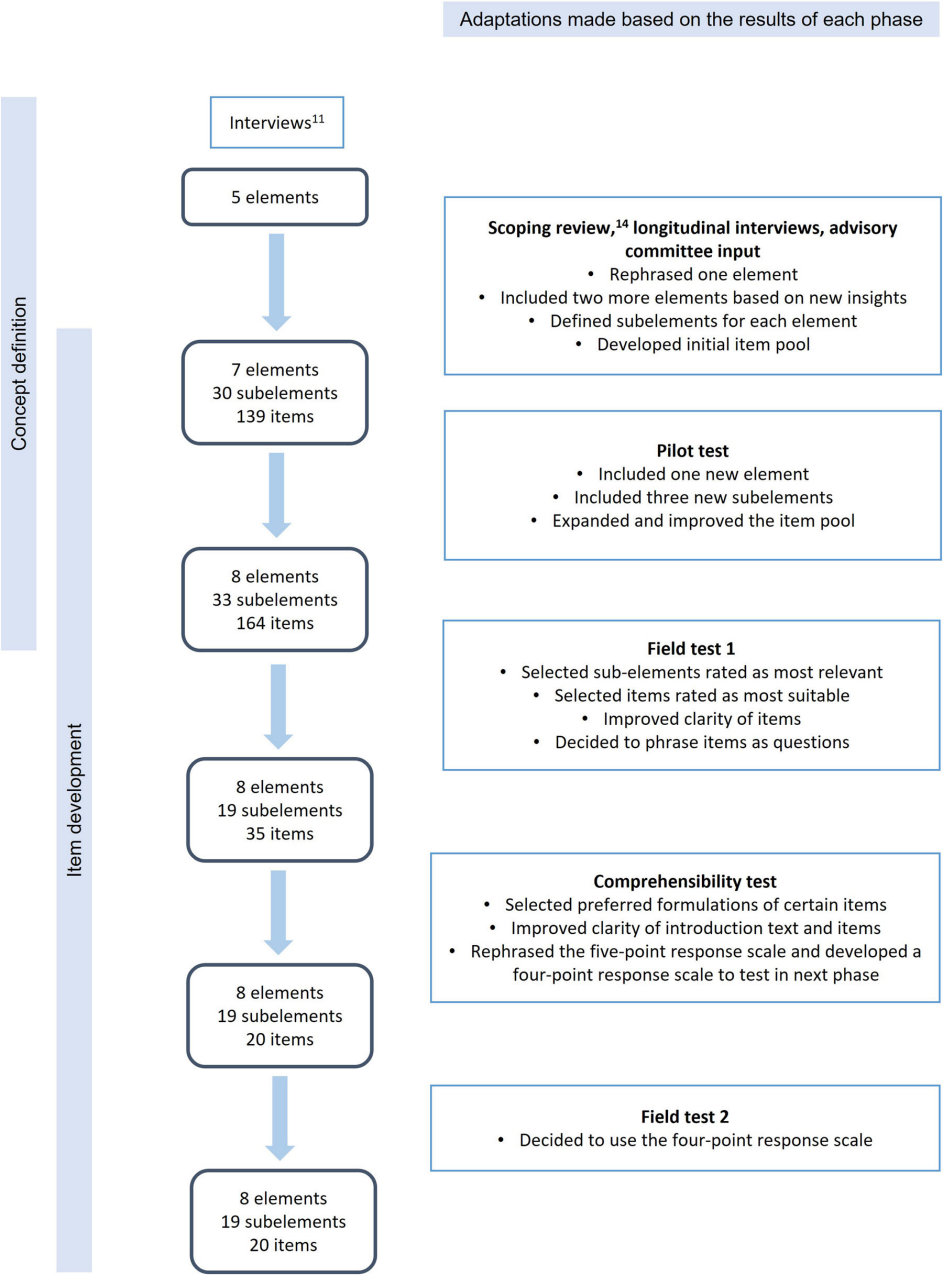
Conceptualisation and questionnaire development process.

The conceptualisation and questionnaire development process is depicted in Figure 1.

### 3.1 Sample characteristics

In total, we included 239 (former) patients, seven adults with limited literacy, five professionals (a medical oncologist, geriatrician, pulmonologist, and two healthcare communication experts; Table 1).

**Table 1:** Participant characteristics.

	Longitudinal interviews	Pilot test	Field test 1	Comprehensibility test	Field test 2	
					Total	Content validity
(Former) patients (*N*)	7	3	152	–	77	61
Age (years)	Md = 67 Range: 56–75	–	*M* = 61.9 SD = 10.5 Range: 32–82		*M* = 59.2 SD = 11.8 Range: 28–82 (1 missing, based on *n* = 76)	*M* = 58.1 SD = 12.2 Range: 28–78
Female (*n*)	4	2	77		45	36
Education level^a^ (*n*)		–				
Low	5		10		8	6
Medium	1		43		27	21
High	1		97		42	34
Missing	–		2		–	–
Type of cancer diagnosis^b^ (*n*)						
Brain	–	–	4		7	2
Breast	2	–	32		24	17
Digestive tract	–	2	24		12	9
Female genital organs	–	–	14		9	9
Haematological	2	–	19		7	6
Head and neck	–	1	7		1	1
Lung	1	–	12		7	7
Male genital organs	–	–	31		10	9
Skin	–	–	6		4	1
Urinary tract	2	–	16		1	1
Other^c^	–	–	11		1	1
Professionals (*N*)	–	5	–	–		–
Adults with low literacy (*N*)	–	–	–	7		–
Age (years)				Range: 30–76		
Female (*n*)				5		

Abbreviations: *M*, mean; Md, median; SD, standard deviation.

^a^ Classified according to Statistics Netherlands, Education categorisation standard 2021.^27^

^b^ Participants could select more than one type of cancer diagnosis.

^c^ Diagnoses selected by less than five participants in total were categorised as ‘Other’.

### 3.2 Conceptualisation and item pool creation

We conducted longitudinal interviews with seven patients, of whom we interviewed six at three time points and one at two time points; the patient had passed away at the time of the third interview. We did not identify any new themes in the last three interviews.

Overall, the results of the longitudinal interviews were in line with the results of our previous qualitative study.^11^ The complete code list can be found in Appendix SD. We identified a sixth element of patient readiness: *self‐efficacy*, that is, the extent to which patients believe they are capable of participating throughout the decision‐making process. This element had not been identified in the previous interview study.

Look, some people will absolutely not want it […] people do not, they do not feel capable of that either. Also not competent. Or afraid they'll make mistakes or ask silly questions. (P703, female patient, third interview)

We had previously identified emotional distress as a factor that could affect various elements of readiness.^11^ In the longitudinal interviews, participants further emphasised the role of emotional distress throughout the SDM process. Emotional distress can make it difficult to be involved and for patients to express themselves.

Especially if I compare myself now to [myself] in the beginning … in the beginning you get such a slap on the head, for me it was really from one minute to the next, then you are just kind of numb. Then you may not always ask the right questions right away. (P703, female patient, third interview)

Emotional distress may also hinder a patient's ability to make a proper decision.

And if you are in a better emotional position, you can also make a better treatment decision. If you're just completely [emotionally] paralyzed and you think ‘whatever, it just has to be done’. That's not entirely fair of course. (P706, female patient, first interview)

Being involved in SDM can also be experienced as difficult and can cause emotional distress. The importance of emotions in patient readiness was also highlighted in our scoping review of qualitative research.^14^ We therefore decided to include *emotional distress* as a seventh element.

The importance of experienced time was further emphasised and clarified. Participants described needing an adequate amount of time. They described needing sufficient time for processing their diagnosis, talking to the clinician, and considering the options. Some participants also mentioned that having too much time to consider the decision can make it more difficult to decide.

And then you are already three weeks further and in those three weeks that uncertainty and fear and ‘what should I do’, so actually deciding, is getting more and more difficult. Then you get more and more in your head, more and more, yes then you become more and more insecure. (P706, female patient, second interview)

We integrated the findings of the previous studies,^11^, ^14^ the longitudinal interviews, and the feedback from advisory committee members on a provisional list of (sub‐)elements. At the end of this phase, we drafted a list of seven elements (i.e., the five previously identified elements, self‐efficacy, and emotional distress) and 30 subelements. We then created an initial item pool. We formulated 4–10 items per subelement, leading to a total of 139 items. We aimed to develop short and comprehensible items.

### 3.3 Pilot test

All patient representatives and professionals (*N* = 8) who were approached completed the pilot test. Based on their feedback, some items were reworded, removed, or added. We further considered the results of all previous steps combined, and decided to include *experienced time* as a new element, rather than as a factor that can influence elements of readiness. We included a total of three more subelements. The list of (sub‐)elements can be found in Appendix SE. The total item pool consisted of 164 items.

### 3.4 Field test 1

We presented the eight elements and 33 subelements to participants in an online survey. A total of 152 participants completed the entire survey. There was no difference in perceived clarity between items formulated as statements or questions (*X* ^2^ (1, *N* = 152) = 1.29, *p* = .26; *X* ^2^ (1, *N* = 152) = 0.11, *p* = .75; *X* ^2^ (1, *N* = 152) = 0.03, *p* = .87). Based on our predefined rules we decided to keep the items phrased as questions, as this is considered to be easier to understand for people with limited (health) literacy.^28^

The relevance of each subelement and corresponding draft items was rated by 49–54 participants. Decisions were made based on pre‐defined rules (Appendix SC), comments from participants, and extensive discussions (S. K. and A. P.). Final decisions were made in consensus. In this phase, some subelements were reworded, grouped, or removed, leading to a final list of 19 subelements (Box 1; Appendix SE). We developed a draft questionnaire with a five‐point response scale (not at all, a little, for a large part, almost completely, completely). We aimed for one item per subelement, with the exception of the subelement ‘The patient understood the probabilities of benefits and risks’, for which we developed two separate items regarding benefits and risks of treatment options. At this stage, we still included multiple formulations for certain items to be tested in the comprehensibility test.

BOX 1. **Subelements of Patient Readiness for shared decision making (SDM) about treatment that are included in the Ready^SDM^**

**Understanding of and attitude towards SDM**

The patient understood that his/her opinion was important.

The patient was open to participation.

**Information skills**

The patient understood the information about the different treatment options.

The patient understood the probabilities of benefits and risks.

The patient could find more information if desired.

**Skills in communicating and claiming space**

The patient dared to express him/herself.

The patient could listen to what the clinician was saying.

The patient dared to ask questions.

The patient felt that what he/she had to say mattered.

**Self‐awareness**

The patient was aware of his/her values and preferences.

The patient was aware of his/her needs in the decision‐making process.

**Consideration skills**

The patient could envision the consequences of potential treatment options for his/her personal life.

The patient was able to compare the different treatment options.

**Self‐efficacy**

The patient felt capable of being involved in SDM.

**Emotional distress**

The patient felt sufficiently calm.

The patient was not too afraid to receive information about the treatments and their consequences.

The patient did not experience involvement in SDM as too much of an emotional burden.

**Experienced time**

The patient experienced sufficient time to talk to the clinician.

The patient experienced an appropriate amount of time to think about the options.

### 3.5 Comprehensibility test

Cognitive interviews were conducted with seven participants with low literacy. Participants rated the clarity of the draft questionnaire with an average of 6.7 on a scale from 1 (not at all comprehensible) to 10 (very comprehensible). Suggested improvements to the introduction text included shortening sentences, clarifying phrases, and leaving out complicated words (such as ‘diagnosis’). Participants provided feedback on the individual items and gave suggestions for enhancing clarity, such as, where possible, using the word ‘choice’ rather than ‘decision’. Participants were presented with multiple formulations for certain items and chose their preferred option. The suggested five‐point scale was not clear to everyone. Some participants did not understand the difference between ‘for a large part’ and ‘almost completely’. They recommended a response scale with fewer options to ensure the difference between the options would be bigger. Specifically, they recommended a three‐point scale.

We created a new version of the questionnaire based on the suggested improvements. We decided against a three‐point response scale, as the recommended minimum number of response options for a valid and reliable questionnaire is four.^29^ We therefore developed a provisional four‐point (not at all; a little; for a large part; absolutely) scale and a rephrased five‐point (not at all; hardly; partly; for a large part; absolutely) scale.

### 3.6 Second field test

In total, 77 participants completed the provisional Ready^SDM^. Thirty‐three participants were randomised to the four‐point response scale, 44 participants to the five‐point response scale. The four‐point response scale showed similar ceiling effects and only slightly lower variability compared to the five‐point scale (Appendix SF). As in the comprehensibility testing phase it was recommended to use a response scale including fewer than five options, we decided to include the four‐point scale in the final questionnaire. Participants who also completed the content validity questions (*n* = 61) were generally positive about the content and clarity of the questionnaire. No items were added, reworded, or removed based on the results of the content validity assessment.

### 3.7 Final questionnaire

The final selection of the subelements that are included in the questionnaire is depicted in Box 1. The final version of the Ready^SDM^ is depicted in Box 2. Each item is scored on a four‐point scale, ranging from ‘not at all’ (0) to ‘absolutely (3). As we assume a formative measurement model, it is appropriate to report scores per element. Element scores can be calculated by taking the average of the items of the relevant element (range: 0–3). Total scores could be calculated by taking the sum of the element scores (range: 0–24).

BOX 2. **Ready^SDM^**

**Participating in the decision‐making process about cancer treatment**

You and your doctor or nurse have made a choice together concerning how to treat your cancer.

You will receive a questionnaire on the next page. It deals with what it was like for you to participate in the decision‐making process about your treatment.

When a patient has cancer, there are often different treatment options to choose from.

- Treatments to cure the disease.
- Treatments to reduce the symptoms.
- Or a monitoring‐only treatment with regular check‐ups.

Please read the questions carefully. Consider all the conversations and information you have had about the possible treatments, and the time you had to think about these. For each question, tick the answer that fits best. There are no right or wrong answers. It's your opinion that counts.

Your answers will remain anonymous. Only the researchers of this questionnaire will see your answers. Your answers will not be shared with your doctor or nurse.

**Topic 1: Making the decision together**

1. Did you know that you could participate in the decision‐making process about which treatment was best for you?
  **Table:** 

Not at all	A little	For a large part	Absolutely
□	□	□	□
2. Did you want to participate in the decision‐making process about the different treatment options?
**Topic 2: Information about the treatments**
3. Did you understand the information about the different treatments available to you?
4. Did you know where to find or get more information if you wanted it?
5. Did you understand how likely the treatments were to work?
6. Did you understand how likely the treatments were to cause side effects?
7. Did you fully understand what the consequences of the different treatments could be for you?
8. Were you able to compare the different treatment options?
**Topic 3: Conversations with the doctor or nurse**
9. Were you able to follow the conversation with the doctor/nurse?
10. Were you able to ask all your questions?
11. Did you feel safe enough to say everything to the doctor/nurse?
12. Did you feel that what you had to say was important?
13. Did you have enough time to talk to the doctor/nurse?
**Topic 4: Feelings**
14. Did you feel calm enough to be able to participate in the decision‐making process?
15. Did you find it unpleasant to participate in the decision‐making process?
16. Did you also want to know about any unpleasant aspects of the different treatment options?
**Topic 5: Your preferences and wishes**
17. Did you know what was important to you in the decision about the treatment?
18. Was the amount of time you had to think about it appropriate for you?
19. Did you know what you needed to be able to participate in making the decision about the treatment? (e.g., time to think, information, or to talk to someone about it)
20. Did you feel that you could participate in making the decision?

## 4 DISCUSSION

Patients generally prefer to be involved in SDM,^2^, ^3^, ^4^ but may not always be ready to be fully involved throughout the decision‐making process. We conducted a thorough process to conceptualise patient readiness for SDM about treatment, and to develop a questionnaire for research to assess readiness in patients with cancer looking back on a decision process. The questionnaire aims to provide better insight into ways to support patients in SDM, and thereby to enhance SDM in daily practice.

Our final conceptualisation of patient readiness consists of both the five elements as previously identified,^11^ with small adjustments in names and subelements based on new insights, and three new elements. First, we included self‐efficacy as a new element. A patient's belief in their capability of participating in SDM is considered part of what makes that a patient is ready to be involved.^12^, ^13^, ^14^ Self‐efficacy may change throughout the process, where for example, positive experiences with sharing thoughts and values may increase patient's self‐efficacy to be involved.^13^

Second, we included two elements that we had previously identified as characteristics that may *affect* specific elements^11^ rather than elements by themselves: emotional distress and experienced time. Emotional distress was previously described to affect the extent to which patients can absorb information, communicate effectively, consider the options, and feel motivated for SDM. Emotions may also help patients in becoming clear on what matters to them.^11^ In the longitudinal interviews and scoping review,^14^ the role of emotional distress was further clarified. Emotional distress can inhibit patients from being able to think about the decision and therefore to be involved. Patients are likely to experience distress due to their diagnosis, and some patients may experience distress from being expected to participate in SDM.^14^ To be involved in SDM, patients may need support to cope with these emotions during the decision‐making process.

Next, experiencing insufficient time is one of the most commonly reported barriers for SDM.^30^ Patients need time to communicate with their clinician, to become informed, and to consider the options.^11^, ^12^, ^14^ At the same time, the longitudinal interviews showed that some participants also experienced *too much* time, as they experienced anxiety and uncertainty while waiting to discuss the decision and for their treatment to commence. Patients may need support in asking for the time they need, and for taking an appropriate amount of time to consider the options. Clinicians can play a role in reducing perceived time pressure, for instance, by making the patient feel that they are the centre of their attention during consultations.^31^ Further, it may help to openly discuss the amount of time that is available to make the decision, as well as how much time the patient needs and wants to consider the options.

Readiness likely fluctuates over time throughout the decision‐making process. The questionnaire aims to retrospectively measure a patient's *overall* level of readiness throughout the entire decision‐making process, and is thus designed to be administered shortly after making the decision. Use of the questionnaire in research may provide new insights into what aspects of readiness patients struggle most with in general. It can further help to identify patient‐, decision‐, clinician‐, and healthcare context‐related factors^15^ that may affect the elements of readiness in different stages of the decision‐making process, both in‐ and outside of clinical encounters. This may be useful to identify support needs of patients, and to identify ways for clinicians to detect and enhance patient readiness in consultations, and thereby SDM in daily practice. The Ready^SDM^ retrospectively assesses the eight identified elements of readiness, capturing a wide variety of self‐perceived skills, cognitions, and emotions. Decision aids can be useful to support patients in elements such as information and consideration skills. Patients who struggle with other elements of readiness, such as emotional distress or communication skills, likely need additional or different support to become more ready for SDM.

### 4.1 Strengths and limitations

We used a multifaceted approach to conceptualise patient readiness and develop the Ready^SDM^. The conceptualisation of readiness was based on previous research complemented with new data. We interviewed patients with cancer at three time points during and after the decision‐making process. This allowed us to get a better understanding of their experiences and needs throughout the process, and it shed further light onto how readiness may fluctuate over time. These longitudinal interviews allowed participants and interviewers to get to know each other, and for patients to reflect on the questions in between interviews. This may have enriched their responses. We then conducted multiple rounds of data collection to develop a questionnaire that is comprehensible, relevant, and comprehensive. To ensure the Ready^SDM^ is suitable for a large group of patients, we tested the comprehensibility among a group of adults with low literacy, and adapted the items based on their feedback.

Our results should also be considered in light of some limitations. First, though we had aimed to include patients with different educational backgrounds, most participants in the field tests had a high educational background. This may have resulted in biases towards what elements and items participants considered most important. Second, we only included complete responses in the first field test, meaning that we may have lost some relevant information. Third, in the second field test we included patients who had faced a decision in the past year. After nearly a year participants may not have fully remembered the decision‐making process anymore, and their answer may have been affected by experiences in the meantime.

### 4.2 Clinical and research implications

Clinicians should be aware that patients may not always be ready to be involved in SDM about treatment. This does not mean that SDM is not possible or should not be attempted, but that patients may need support in the various demands that SDM imposes on them. Patients may need support for one or more of the elements of readiness. For instance, patients may need support in becoming clear on their needs in the decision‐making process, expressing their concerns and questions, and coping with emotional distress. Support needs likely differ between patients. Within patients, support needs may also differ between and within decision‐making processes. The Ready^SDM^ has the potential to provide better insight into what areas of readiness patient struggle most with, and to detect potential associations with patient‐ and context‐related characteristics. Before using the questionnaire, further research needs to be conducted to determine its psychometric properties. Further, the construct of readiness was defined regardless of diagnosis, yet the questionnaire has been developed in an oncological setting. The questionnaire is likely to be relevant for other types of treatment decisions as well. Still, it is possible that there are differences in what subelements patients find most relevant for the assessment of readiness in other settings. Therefore, before using the Ready^SDM^ in other settings, it is recommended to first conduct a field test among the target population to determine content validity.

## 5 CONCLUSION

A variety of elements contribute to the extent to which patients feel ready to be involved in SDM about treatment. Readiness is expected to vary within patients across the decision process. The Ready^SDM^ could be used in research to study which elements of readiness (subgroups of) patients with cancer encounter the most challenges with, and to identify their support needs. Further research needs to be conducted to determine its psychometric properties.

## AUTHOR CONTRIBUTIONS

**Sascha M. Keij**: Writing—original draft; methodology; formal analysis; investigation; data curation; project administration. **Anne M. Stiggelbout**: Funding acquisition; writing—review and editing; methodology; supervision; conceptualisation. **Arwen H. Pieterse**: Funding acquisition; writing—review and editing; methodology; supervision; conceptualisation; data curation; investigation; formal analysis; project administration.

## CONFLICT OF INTEREST STATEMENT

The authors declare no conflicts of interest.

## ETHICS STATEMENT

The Medical Ethical Committee of the Leiden University Medical Center (LUMC) provided exemption for ethical approval of the study protocol (P17.121), according to Dutch Law.

## Supporting information

Supporting information.

Supporting information.

Supporting information.

Supporting information.

Supporting information.

Supporting information.

## Data Availability

The data that support the findings of this study are available from the corresponding author upon reasonable request.
