# Correction to “Patient Readiness for Shared Decision Making About Treatment: Conceptualisation and Development of the Ready^SDM^”

**DOI:** 10.1111/hex.70843

**Published:** 2026-08-25

S. M. Keij, A. M. Stiggelbout, and A. H. Pieterse, “Patient Readiness for Shared Decision Making About Treatment: Conceptualisation and Development of the Ready^SDM^,” *Health Expectations* 27, no. 2 (2024): e13995.

In Section 3.7, we describe the final questionnaire and the scoring of each item. Each item is scored on a four‐point scale, ranging from ‘not at all’ (0) to ‘absolutely’ (3). Due to an error, part of the description of the scale was omitted. The correct text should read as follows:

The final selection of the subelements that are included in the questionnaire is depicted in Box 1. The final version of the Ready^SDM^ is depicted in Box 2. Each item is scored on a four‐point scale, ranging from ‘not at all’ (0) to ‘absolutely’ (3). Item 15 (‘Did you find it unpleasant to participate in the decision‐making process?’) should be **reverse coded**. That is, lower scores should be recoded into higher scores and vice versa (e.g., score 0 becomes score 3). Reverse coding is only necessary for item 15. As we assume a formative measurement model, it is appropriate to report scores per element. Element scores can be calculated by taking the average of the items of the relevant element (range: 0–3). Total scores could be calculated by taking the sum of the element scores (range: 0–24).

We apologize for this error.

