# Supplementary figures and images for: Patient readiness for shared decision making about treatment: Conceptualisation and development of the ReadySDM
Source: Health Expect. 2024 Feb 23;27(2):e13995. doi: 10.1111/hex.13995 (PMC10891436; doi:10.1111/hex.13995)

## Process of ‘making decisions together’

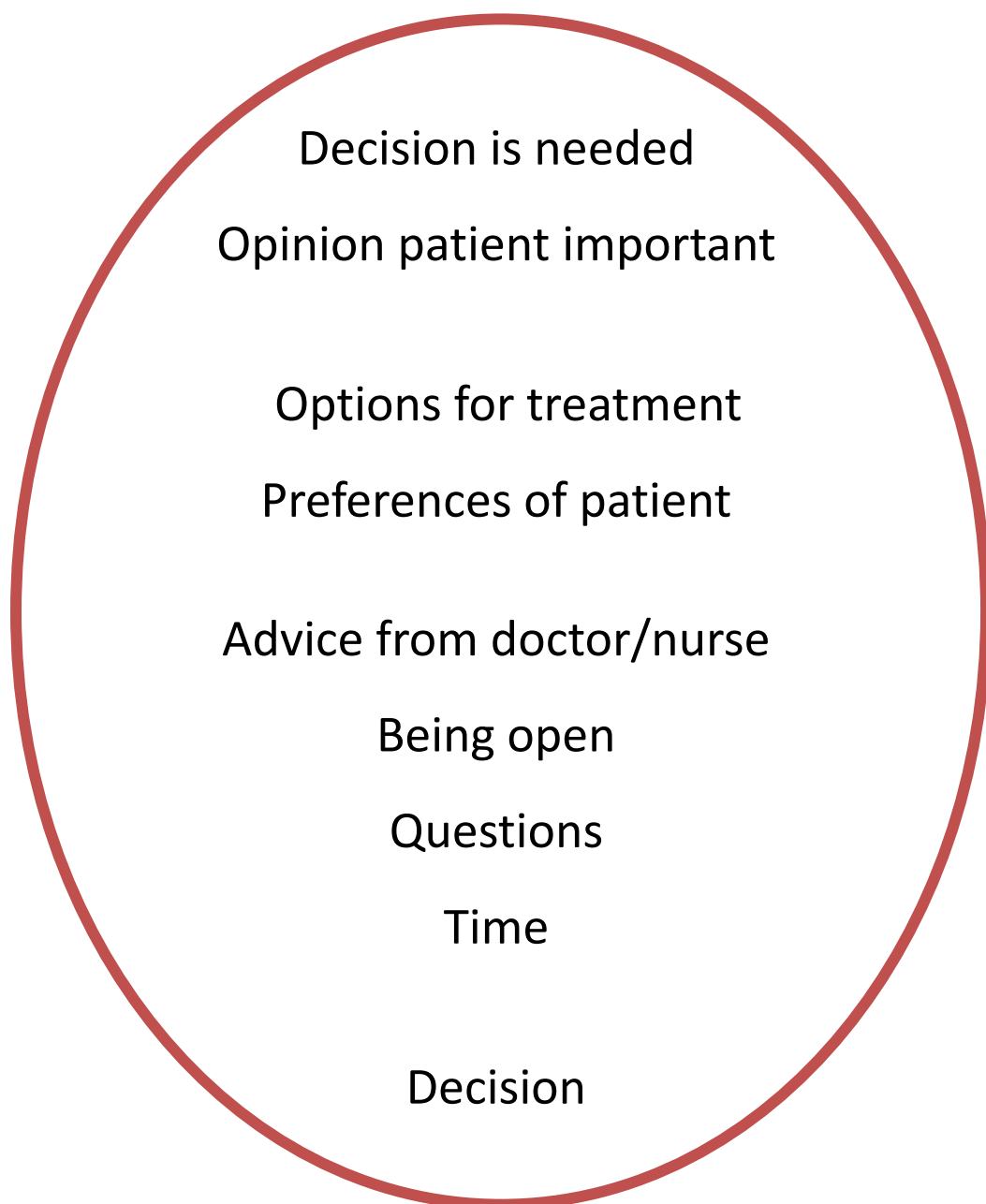

Supplement: Supplementary file 1 — Supporting information. [file HEX-27-e13995-s001.pdf]
