# Supplementary material for: Patient readiness for shared decision making about treatment: Conceptualisation and development of the ReadySDM
Source: Health Expect. 2024 Feb 23;27(2):e13995. doi: 10.1111/hex.13995 (PMC10891436; doi:10.1111/hex.13995)
Supplement: Supplementary file 2 — Supporting information. [file HEX-27-e13995-s003.pdf]

Appendix SB. Interview Guides Longitudinal interviews

<u>**Interview 1**</u>

- Ask if there is anyone (e.g., partner, children, other family member, friends) who will be involved in the decision-making process, and if so, who.
- Questions about disease: diagnosis and date of diagnosis.
- Ask if patient has an idea about the type of treatment, aim of treatment, and between which treatment options a decision will have to be made. Ask what the patient expects of the next consultation with the clinician, and what the aim of the consultation is.

Have you ever heard of ‘making decisions together’?

a. If yes: How would you describe ‘making decisions together’?

b. If not: What do you think ‘making decisions together’ could be?

When a patient has cancer, there may different ways to treat the disease. When a patient decides together with the doctor about which treatment is best suited for the patient, we call this ‘making decisions together’. Making decisions together often consists of the following steps.

- *Firstly, the doctor tells the patient that a decision has to be made about the treatment. And that the opinion of the patient is important to make this decision.*
- *Then, the doctor gives information about which treatment options are available. This can also be the option of no treatment. The doctor also gives information about the advantages and disadvantages of these options.*
- *Then, the doctor and patient talk about what the patient thinks of the options, and they discuss the preferences of the patient.*
- *Finally, the doctor or the patient decides which treatment is best suited for the patient.*

So making decisions together about treatment is a process. In this process the doctor and patient search for what treatment fits best in the life of the patient. This process starts when the patient knows that a decision has to be made about the treatment. And the process ends when that decision has been made. All of this can happen during one consultation. But for some decision this takes more time. The patient can then have multiple appointments with the doctor or nurse, or take the time to think about the decision and talk about it with others.

Patients may also want to get an advice from the doctor. And it can be important that the doctor gets to know the patient, and that patients freely discuss their thoughts and feeling.

Soon a decision has to be made about your treatment. We would like to know what patients need to be ready to make decision about treatment together with their doctor or nurse.

1. Patients and doctors sometimes say that 'making decisions together' can sometimes be difficult for patients. Why do you think that 'making decisions together' is sometimes difficult for patients?
    a. What makes that 'making decisions together' is sometimes difficult for patients?
    b. What could make that 'making decisions together' would be easier for patients?
2. What must the patient be able to do to 'making decisions together' with a doctor or a nurse?
    a. What would make that a patient is ready for 'making decisions together'?
3. What else is needed for them?
4. Patients sometimes see more than one doctor who is involved in treatment of the disease. Is that the case in your situation?
    a. Do you think that having more than one doctor makes it more or less difficult for patients to be involved in 'making decisions together'?
    b. How could having more than one doctor make it more difficult for patients to be ready for 'making decisions together'?
    c. How could having more than one doctor make it easier for patients to be involved in 'making decisions together'?
5. What do you think could be done to make it more likely that patients can participate in 'making decisions together'?
    a. Why would that work?
    b. Is there anything patients can do to make "making decisions together' more likely to happen?
        i. What?
        ii. Why would that work?
6. Any additional points?

The description of SDM provided at the beginning of the interview is the same as we provided in a previous interview study by our research group.[11] Some questions of the topic guide were also based on the topic guide of this previous study.

Interview 2

- Ask what decision has been made about the treatment, what the aim of the treatment is, and between which treatments a decision has been made.
- Ask if there is anyone (e.g., partner, children, other family member, friends) who was involved in the decision-making process, and if so, who.

During the first interview I described ‘making decisions together’. Would you like me to repeat that description?

[If the answer is yes, repeat the following steps:]

- *Firstly, the doctor tells the patient that a decision has to be made about the treatment. And that the opinion of the patient is important to make this decision.*
- *Then, the doctor gives information about which treatment options are available. This can also be the option of no treatment. The doctor also gives information about the advantages and disadvantages of these options.*
- *Then, the doctor and patient talk about what the patient thinks of the options, and they discuss the preferences of the patient.*
- *Finally, the doctor or the patient decides which treatment is best suited for the patient.*

Recently a decision about your treatment has been made. We would like to know what patients need to be ready to make decision about treatment together with their doctor or nurse.

1. In our last conversation we talked about that patients and doctors sometimes say that ‘making decisions together’ can sometimes be difficult for patients.
    a. You then said that [briefly list key findings from first interview] can make it difficult for patients to decide together with their doctor. It is now [name how long it has been] later and a decision has been made about your treatment. What would you now say that can make that ‘making decisions together’ is sometimes difficult for patients?
    b. You then said that [briefly list key findings from first interview] can make it easier for patients to decide together with their doctor. It is now [name how long it has been]

later and a decision has been made about your treatment. What would you now say that can make that 'making decisions together' easier for patients?

2. You previously that the patient should be able to [briefly list key findings from first interview] to make decisions together with the doctor or nurse. How do you feel about that now?
    a. What would make that a patient is ready for 'making decisions together'?
3. What else is needed for patients?
4. Patients sometimes see more than one doctor who is involved in treatment of the disease. Is that the case in your situation right now?
    a. You previously said that having more than one doctor can make it more difficult for patients to be ready for 'making decisions together] because [briefly list key findings from first interview]. How do you feel about that now?
    b. You previously said that having more than one doctor can make it easier for patients to be ready for 'making decisions together' because [briefly list key findings from the first interview]. How do you feel about that now?
5. I asked you last time what could be done to make it more likely that patients can participate in 'making decisions together'. You then said that [briefly list key findings from the first interview] would make it more likely. How do you feel about that now?
    a. Why might that work?
    b. You mentioned earlier that patients can do [briefly list key findings from first interview] to make it more likely that they can decide with the doctor. How do you feel about that now?
        i. What could work?
        ii. Why would that work?
6. Any additional points?

Interview 3

During the first interview I described ‘making decisions together’. Would you like me to repeat that description?

[If the answer is yes, repeat the following steps:]

- *Firstly, the doctor tells the patient that a decision has to be made about the treatment. And that the opinion of the patient is important to make this decision.*
- *Then, the doctor gives information about which treatment options are available. This can also be the option of no treatment. The doctor also gives information about the advantages and disadvantages of these options.*
- *Then, the doctor and patient talk about what the patient thinks of the options, and they discuss the preferences of the patient.*
- *Finally, the doctor or the patient decides which treatment is best suited for the patient.*

Recently a decision about your treatment has been made. We would like to know what patients need to be ready to make decision about treatment together with their doctor or nurse.

1. In our previous conversations we talked about that patients and doctors sometimes say that ‘making decisions together’ can sometimes be difficult for patients.
    a. You then said that [briefly list key findings from previous interviews] can make it difficult for patients to decide together with their doctor. It is now a few months after a decision has been made about your treatment. What would you now say that can make that ‘making decisions together’ is sometimes difficult for patients?
    b. You previously said that [briefly list key findings from previous interviews] can make it easier for patients to decide together with their doctor. It is now a few months after a decision has been made about your treatment. What would you now say that can make that ‘making decisions together’ easier for patients?
2. You previously that the patient should be able to [briefly list key relevant findings from previous interviews] to make decisions together with the doctor or nurse. How do you feel about that now?
    a. What would make that a patient is ready for ‘making decisions together’?
3. What else is needed for patients?

4. Patients sometimes see more than one doctor who is involved in treatment of the disease. Is that or has that been the case for you?
    a. You previously said that having more than one doctor can make it more difficult for patients to be ready for ‘making decisions together] because [briefly list key findings from previous interviews]. How do you feel about that now?
    b. You previously said that having more than one doctor can make it easier for patients to be ready for ‘making decisions together’ because [briefly list key findings from previous interviews]. How do you feel about that now?
5. I previously asked you what could be done to make it more likely that patients can participate in ‘making decisions together’. You then said that [briefly list key findings from the previous interviews] could make it more likely. How do you feel about that now?
    a. Why might that work?
    b. You mentioned earlier that patients can do [briefly list key findings from previous interviews] to make it more likely that they can decide with the doctor. How do you feel about that now?
        i. What could work?
        ii. Why would that work?
6. Any additional points?
