# Supplementary material for: Patient readiness for shared decision making about treatment: Conceptualisation and development of the ReadySDM
Source: Health Expect. 2024 Feb 23;27(2):e13995. doi: 10.1111/hex.13995 (PMC10891436; doi:10.1111/hex.13995)
Supplement: Supplementary file 3 — Supporting information. [file HEX-27-e13995-s005.pdf]

Appendix SC. Pre-defined decision rules results field tests

Field test 1

Decision criteria to include or exclude particular subelements in the questionnaire

1. The participants consider the subelement part of those most important for a particular element:
    a. >50%: include
    b. 25-50%: discuss whether to include
    c. <25%: strongly consider to exclude
2. The participants consider the subelement not important:
    a. >50%: exclude
    b. 25-50%: strongly consider not to include
        i. If also rated by <25% as most important subelement: definitely exclude
3. Consider all suggestions in comments. If needed, discuss with project group members.
4. All final decisions are made in consensus between Sascha Keij and Arwen Pieterse.

Decision criteria about which items to include in the questionnaire

Note: in this phase we can include multiple items per subelement, and test in the next phase which one is most comprehensible.

1. The participants consider the question part of those most appropriate:
    a. >50%: strongly consider to include
    b. 25-50%: discuss whether to include
    c. <25%: exclude
2. Consider all suggestions in comments. If needed, discuss with project group members.
3. All final decisions are made in consensus between Sascha Keij and Arwen Pieterse.

Items phrased as questions versus statements

1. Test if there is a significant preference for questions or statements
    a. Preference for questions: keep items formulated as questions.
    b. No preference: keep items formulated as questions, as this has also been suggested by Pharos to improve comprehensibility.
    c. Preference for statement: consider changing items to statements, test in next phase (comprehensibility testing).

Field test 2

Decision criteria for including or excluding items

1. The participants would exclude a particular item:
    a. >50%: exclude
    b. 25-50%: discuss whether to exclude
    c. <25%: do not exclude
2. Consider all suggestions in comments. If needed, discuss with project group members.
3. All final decisions are made in consensus between Sascha Keij and Arwen Pieterse.
