# Supplementary material for: Patient readiness for shared decision making about treatment: Conceptualisation and development of the ReadySDM
Source: Health Expect. 2024 Feb 23;27(2):e13995. doi: 10.1111/hex.13995 (PMC10891436; doi:10.1111/hex.13995)
Supplement: Supplementary file 4 — Supporting information. [file HEX-27-e13995-s004.pdf]

Appendix SD. Coding structure

Note: the coding structure consists of codes and categories from a previous qualitative study[11] supplemented with new codes and categories from the longitudinal interviews.

Categories	Subcategories	Codes
**Understanding of SDM process**	**Understanding and awareness of choice**	33. Understanding that there is no best option<br>142. Not perceiving choice
	**Understanding of (role in) SDM**	10. Understand that/why express/give opinion<br>11. Understand that/why need to agree with decision<br>15. Understand that/why role in decision making process<br>46. Understand SDM process<br>49. Knowledge of healthcare practice<br>61. Awareness that a decision needs to be made/there is a choice
	**Equality and paternalistic expectations**	13. (Not) feeling equal<br>64. Paternalistic expectations clinician
	**Attitude and needs**	37. Feeling autonomous<br>58. Trust in care<br>59. Open attitude<br>65. Need for control<br>95. No active role patient<br>102. Patient needs to be motivated<br>107. Patient in 'action' mode<br>111. Patient thinks along actively
**Information skills**	**Information: understanding, knowledge, application**	7. Understanding of chances/risks<br>18. Repeating of information<br>19. Simple explanations needed (no jargon)<br>23. Understanding information<br>24. Uninformed preferences<br>28. Cognitive overload

		44. Processing and applying of information<br>50. Dosed information/tools<br>63. Basic understanding needed health/illness<br>100. Afraid that information is too difficult<br>128. Daring to express irritations<br>138. Remembering information
	**Information: searching**	76. Finding information yourself<br>77. Digital
	**Information: needs**	31. Specific/personalized information<br>39. Concrete information<br>53. Need for honest information<br>62. Information on same level<br>66. Need for information<br>67. Consistent information<br>72. Clear information options<br>78. Receiving sufficient information<br>93. Giving treatment options (clinician)<br>101. Being given information<br>126. Patient does not want statistics<br>127. Clinician gives justification<br>136. Medical interest
**Communication skills and claiming space**	**Express**	12. Assertiveness<br>20. Verbalize thoughts<br>30. Feeling free to express<br>40. Express boundaries<br>41. Asking for needed time<br>84. Expressing values<br>97. Formulate needs<br>122. Worried to be whining<br>131. Clinician gives patient opportunity to express thoughts

		132. Clinician asks opinion of patient/significant other<br>133. Express emotions<br>144. Sharing personal information
	**Ask questions**	57. Dare to ask questions<br>74. Ask questions
	**Communication clinician-patient**	80. Listening (patient)<br>89. Clinician and patient same steps in process<br>90. Thinks along actively (clinician)<br>91. Understanding of clinician<br>94. Clinician waits with giving advice
**Self-awareness**	**Insight own preferences and values**	34. Insight in values<br>42. Being aware of 'boundaries'<br>43. Being aware if you want to know certain information<br>108. Clinician aware of thinking process patient
**Consideration skills**	**Overseeing consequences**	86. Overseeing consequences
	**Thinking, considering**	68. Sufficiently being able to consider
	**Opinions and experiences of others**	3. Feeling alone<br>45. Feeling that someone thinks along<br>52. Needing opinion of other<br>73. Needing opinion clinician<br>85. Argumentation preference clinician<br>137. Clinician stimulates patient to consider<br>140. Considering is difficult
**Self-efficacy**		121. (In)secure about own capabilities
**Characteristics that may affect elements**	**Specific groups of patients**	103. Difficult for older patients<br>106. Difficult for lower educated/psychiatric comorbidity/immigrants/low (health) literacy/language barriers
	**Emotions**	8. Experiences emotional distress<br>17. Absorbing less information due to emotions<br>26. Considering/deciding difficult due to emotions

	51. Anticipated regret<br>56. Emotions can help with deciding<br>71. Asking less questions due to emotions<br>112. Patient needs to dare to make a decision<br>134. Being given a choice causes insecurity<br>135. Not expressing thoughts due to emotions<br>139. Deciding is intense<br>141. Indecisiveness makes it harder, not daring to choose<br>143. Needing emotional support
**Acceptance of diagnosis**	55. Accepting diagnosis
**Close relationships**	14. Support from close relationships<br>69. Not too much pressure close relationships<br>96. Taking influence of decision on close relationships into account<br>118. Significant others do not interfere
**Decision characteristics**	105. More SDM when further along in SDM<br>110. Being able to revisit decision<br>120. Uncertainty about illness
**Relationship clinician-patient**	21. Trust in clinician<br>32. Feeling of being listened to<br>75. Good relationship clinician<br>79. Patience of clinician<br>82. Feeling of being taken seriously<br>87. Getting to know patient<br>54. Feeling safe/comfortable<br>119. Clinician does not involve patient<br>130. Multiple clinicians<br>130.1 Seeing multiple clinicians is confusing<br>145. Agree on role of others
**Time**	114. Time: finding information<br>60. Do not express due to time pressure<br>4. Time - talking with clinician

		2. Time: thinking<br>27. Time: processing<br>98. Awareness that taking time does not influence disease process<br>48. Time: relationship development healthcare professional<br>125. Waiting makes you insecure
	**Previous experience**	123. Previous experiences
