# Supplementary material for: Patient readiness for shared decision making about treatment: Conceptualisation and development of the ReadySDM
Source: Health Expect. 2024 Feb 23;27(2):e13995. doi: 10.1111/hex.13995 (PMC10891436; doi:10.1111/hex.13995)
Supplement: Supplementary file 5 — Supporting information. [file HEX-27-e13995-s002.pdf]

Appendix SE. The construct of patient readiness for shared decision making about treatment: selection of elements and subelements.

First selection based on interview study[11]	Selection after incorporating results of scoping review,[14] longitudinal interviews among patients with cancer, and feedback from the advisory committee	Selection after pilot test and further consideration among research team members	Selection to incorporate in the Ready[SDM] in an oncological treatment setting
**Understanding of and attitude towards SDM**	**Understanding of and attitude towards SDM**	**Understanding of and attitude towards SDM**	**Understanding of and attitude towards SDM**
	The patient..	*The patient..*	*The patient..*
	1. understood that there was not one best option	1. understood that there was not one best option	
	2. understood why his/her opinion was important	2. understood why his/her opinion was important	1. understood that his/her opinion was important
	3. was willing to participate (in the decision-making process)	3. was willing to participate in decision making about treatment	2. was open to participation in SDM
	4. was open to consider the possible treatment options	4. was open to consider the possible treatment options	
**Health literacy**	**Information skills**	**Information skills**	**Information skills**
	1. understood information about the options	1. understood the information about the possible treatment options that the doctor/nurse told them about	1. understood the information about the different treatment options
		2. understood the information about the different treatments online/on paper	
	2. was able to apply the information to his/her own situation	3. was able to apply the information to his/her own situation	
	3. was able to remember the information	4. was able to remember the information	
	4. understood information about probabilities	5. understood information about probabilities	2. understood the probabilities of benefits and risks
	5. was able to look for more information if he/she wanted to do	6. was able to look for more information online if he/she wanted to do	3. could find or get more information if desired

	6. was not afraid that information would be too difficult<br>7. did not feel overwhelmed by the amount/complexity of the information	7. was not afraid that information would be too difficult<br>8. did not feel overwhelmed by the amount/complexity of the information<br>9. had sufficient information	
**Skills in communicating and claiming space**	**Skills in communicating and claiming space**	**Skills in communicating and claiming space**	**Skills in communicating and claiming space**
	1. was able to formulate thoughts	1. was able to formulate thoughts	
	2. was able to formulate needs	2. was able to formulate needs	
	3. dared to express him/herself<br>4. dared to express emotions/fears	3. dared to express himself/herself<br>4. dared to say how he/she felt or what he/she was afraid of.	1. dared to express himself/herself
	5. dared to revisit the decision if he/she would have wanted to	5. dared to revisit the decision if he/she would have wanted to	
	6. was able to listen to what the clinician was saying	6. was able to listen to what the clinician was saying	2. was able to listen to what the clinician was saying
	7. dared to ask questions	7. dared to ask questions	3. dared to ask questions
	8. dared to ask follow-up questions	8. dared to ask follow-up questions	
			4. felt that what he/she had to say mattered
**Self-awareness**	**Self-awareness**	**Self-awareness**	**Self-awareness**
	1. was aware of what matters to him/her<br>2. was aware of his/her preferences	1. was aware of what matters to him/her<br>2. was aware of his/her preferences	1. was aware of his/her values and preferences
	3. was aware of his/her needs	3. was aware of his/her needs	2. was aware of his/her needs in the decision-making process
**Consideration skills**	**Consideration skills**	**Consideration skills**	**Consideration skills**
	1. could envision the consequences of potential treatment options for his/her personal life	1. could envision the consequences of potential treatment options for his/her personal life	1. could envision the consequences of potential treatment options for his/her personal life
	2. was able to consider	2. was able to compare the different treatment options	2. was able to compare the different treatment options
	3. was able to cope with uncertainty	3. was able to cope with uncertainty	

**Self-efficacy**	**Self-efficacy**	**Self-efficacy**
1. felt capable of being involved in SDM	1. felt capable of being involved in SDM	1. felt capable of being involved in SDM
2. felt able to contribute to the decision	2. felt able to contribute to the decision	
**Emotional distress**	**Emotional distress**	**Emotional distress**
1. was not too emotional due to the diagnosis	1. was not too emotional	1. felt sufficiently calm
2. was not too afraid to receive information about the treatments and their consequences	2. was not too afraid to receive information about the treatments and their consequences	2. was not too afraid to receive information about the treatments and their consequences
3. did not experience involvement in SDM as an emotional burden	3. did not experience involvement in SDM as too much of an emotional burden	3. did not experience involvement in SDM as too much of an emotional burden
	**Experienced time**	**Experienced time**
	1. experienced an adequate amount of time to be involved in decision making	1. experienced sufficient time to talk to the clinician
		2. experienced an adequate amount of time to think about the options

Gray shading indicates that two subelements were merged, or one subelement was split into two subelements.
