# Supplementary material for: Patient readiness for shared decision making about treatment: Conceptualisation and development of the ReadySDM
Source: Health Expect. 2024 Feb 23;27(2):e13995. doi: 10.1111/hex.13995 (PMC10891436; doi:10.1111/hex.13995)
Supplement: Supplementary file 6 — Supporting information. [file HEX-27-e13995-s006.pdf]

Appendix SF. Response scales

Background characteristics

There were no significant differences in gender, age and educational background between the participants who received the questionnaire with the four- or five-point response scales.

Table 1. Characteristics of participants (N=77) who received the questionnaire with four-point or five-point response scale.

Characteristic	Four-point scale (N, %)	Five-point scale (N, %)
Total	44 (57)	33 (43)
Female gender	26 (59)	19 (58)
Age (M, SD)	58.9 (11.8)	59.7 (12.0)
Educational background		
- low	4 (9)	4 (12)
- medium	16 (36)	11 (33)
- high	24 (55)	18 (55)

Variation

To assess the variation we calculated the sum of item scores. Only complete responses were included. As both scales had a different total range (the four-point scale from 0 to 60; the five-point scale from 0 to 80) we calculated the coefficient of variation (CV), i.e., the ratio of the standard deviation (SD) to the mean (M), calculated as SD/M. Further, to compare the median and range scores, we recalculated these scores to a 0-100 scale.

The CV-score of the five-point scale is higher (.24) than of the four-point scale (.19). The recalculated median and range scores were similar. The skweness and Kurtosis scores of both the four- and the five-point scales indicate a normal distribution.

Table 2. Descriptive characteristics of the four- and five-point response scale.

	Four-point scale	Five-point scale
Missing (n)	0	3
M (SD)	44.8 (8.3)	58.2 (14.3)
CV	.19	.24
Range (min, max)	35 (22, 57)	45 (31, 76)
Median	46	63
Recalculated median (scale 0-100)	76.7	78.8
Recalculated range (scale 0-100)	58.3	56.2
Skewness	-.89	-.66
Kurtosis	.57	-.82

Ceiling effects

To compare ceiling effects, we calculated the percentage of participants who selected the highest response option for each item. As the number of options to choose from, and thus the chance to select the a specific response option, is uneven between the two groups we also calculated an adjusted percentage for the four-point scale to take this into account (calculated as: % of participants who selected the highest score/.25x0.20).

Ceiling effects differed per item. A comparison of the percentage of participants who selected the highest score on the four-point scale to the percentage of participants who selected the highest score on the five-point scale, showed comparable overall ceiling effects. That is, for some items we saw similar ceiling effects (a difference of less than 5%). On other items there were larger differences; for the unadjusted percentage this was more often in favor of the five-point scale, for the adjusted percentage this was more often in favor of the four-point scale.

Table 3. Percentage of participants who selected the highest score per item.

	Four-point scale			Five-point scale	
Item	N	%	Adjusted %	N	%
1	44	50.0	40.0	33	18.2
2	44	70.5	56.4	33	33.3
3	44	54.5	43.6	33	42.4
4	44	52.3	41.8	33	45.5
5	44	36.4	29.1	33	42.4
6	44	31.8	25.4	32	43.8
7	44	27.3	21.8	32	37.5
8	44	22.7	18.2	29	27.6
9	44	68.2	54.5	32	68.8
10	44	79.5	63.6	32	75.0
11	44	79.5	63.6	32	71.9
12	44	59.1	47.3	32	50.0
13	44	61.4	49.1	32	56.3
14	44	52.3	41.8	32	34.4
15[a]	44	84.1	67.3	32	59.4
16	44	81.8	65.4	32	75.0
17	44	43.2	34.6	33	45.5
18	44	52.3	41.8	33	45.5
19	44	54.5	43.6	32	40.6
20	44	59.1	47.3	32	40.6

[a] This item is negatively phrased, so this represents the percentage of participants who selected the lowest score.
